# Keeping up with the ′omics: non-equilibrium models of gene regulation

**DOI:** 10.1186/s12915-015-0117-5

**Published:** 2015-02-10

**Authors:** David Pincus

**Affiliations:** Whitehead Institute for Biomedical Research, Nine Cambridge Center, Cambridge, MA 02142 USA

## Abstract

Non-equilibrium processes are vital features of biological systems. Despite this universally accepted fact, gene regulation is typically formalized into models that assume thermodynamic equilibrium. As experimental evidence expands the repertoire of non-equilibrium genome regulatory mechanisms, theoreticians are challenged to devise general approaches to accommodate and suggest functions for non-equilibrium processes. Ahsendorf *et al*. provide one such framework, which is discussed in the context of the growing complexity of eukaryotic gene regulation.

## Commentary

Life is an excursion away from equilibrium. From the concentration gradients cells establish both with their environments and within themselves, to chaperone-assisted protein folding, energy-consuming cellular processes serve to fend off equilibrium. Such non-equilibrium processes are essential for life: as Ahsendorf *et al.* state in their paper in the current issue of *BMC Biology*, ‘we are only at equilibrium when we are dead’ [1].

The fundamental hallmark of an equilibrium process is reversibility. Biosynthetic processes, however, rely on a combination of reversible, passive steps and irreversible, energy-consuming steps. These irreversible steps allow biological systems to attain arbitrarily high degrees of fidelity, and as such are vital for information processing and propagation [2]. In particular, each step of gene expression relies on non-equilibrium proofreading mechanisms to ensure fidelity: transcription, splicing and translation all rely on both thermodynamic equilibrium steps and irreversible kinetic steps. For example, the initial binding of a tRNA anti-codon to the codon in the decoding center of the ribosome is an equilibrium event, while the GTP hydrolysis-dependent proofreading is a non-equilibrium process required for fidelity [3].

**Figure 1:**
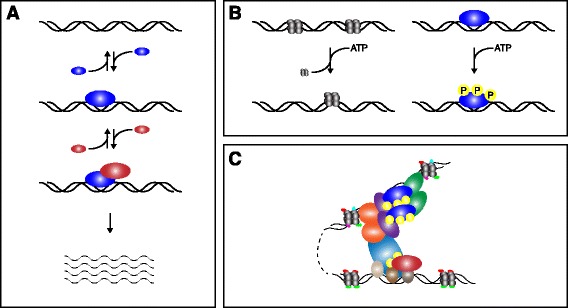
**Equilibrium and non-equilibrium events in gene regulation. (A)** Thermodynamic equilibrium depiction of transcriptional regulation in prokaryotes. A transcription factor (blue) reversibly binds to naked DNA with a given affinity driven by mass action. The bound transcription factor then reversibly recruits RNA polymerase (red) to initiate transcription. **(B)** Examples of non-equilibrium processes involved in eukaryotic gene regulation. On the left, ATP-dependent nucleosome remodeling is shown. On the right, transcription factor phosphorylation is depicted. Such energy-dissipating processes are known to be involved in eukaryotic transcription, though these events are frequently left out of mathematical and computational models of gene regulation. **(C)** Cartoon of a dramatically over-simplified mammalian transcription initiation complex. Nucleosome remodeling and modification, transcription factor clustering in a ‘super-enhancer’, DNA looping, transcription factor and RNA-polymerase phosphorylation and cooperative binding interactions between general transcription factors, mediator, a distal enhancer and RNA polymerase all occur in these dynamic structures that are very far from equilibrium.

Despite our implicit understanding that non-equilibrium mechanisms play pivotal roles in many biological processes, we often explicitly treat eukaryotic gene regulation as if it were an equilibrium process. That is to say, even though we know that ATP-dependent chromatin remodeling, histone acetylation, DNA methylation, DNA looping, RNA polymerase C-terminal domain phosphorylation and other energy-dissipating events are required for transcriptional specificity, fidelity and efficiency, we ignore these ‘details’ and formalize transcriptional activation as a passive process that operates at thermodynamic equilibrium (Figure 1).

There are three main reasons we tend to model gene regulation with an equilibrium framework. The first is historical: the original computational model of gene regulation, which focused on the transcriptional switch at the heart of the lytic/lysogenic fate decision of phage λ, treated transcription factors binding to and dissociating from naked DNA - an equilibrium process - as the function that determined transcriptional output [4]. The equilibrium formalism was perfectly suited to this case of gene regulation and captured the dynamics of the process so well that it has been extended to other gene expression circuits that are far beyond its intended reach.

The second reason is that, despite the complexity of eukaryotic transcription, the equilibrium approximation works quite well. In many circumstances, within the precision of experimental capabilities, the equilibrium model can fit the data. This of course begs the question of whether it is necessary to bother accounting for non-equilibrium processes at all. Moreover, while we can list dozens of non-equilibrium processes that relate to gene regulation, we frequently have no idea which events are important to make explicit in a given situation, what the function of a particular energy-consuming event is or what the cost of neglecting a reaction might be. Thus, in the name of parsimony and because the equilibrium approximation gives results that are good enough to match the level of resolution to which we have experimental data, we ignore the non-equilibrium processes.

The third reason we still rely on equilibrium models is that we lack a straightforward theoretical framework to model non-equilibrium complexity. Without such a framework, we risk relegating the role of models to recapitulation. That is to say, as equilibrium approximations, the chief function of gene regulation models is to test if our assumptions for how a circuit is operating are sufficient to explain the experimental results we observe. If, on the other hand, we would like to use models to gain insight into the functions of aspects of gene regulation that we do not fully understand, such as post-translational modification of transcription factors, then we need methods that accommodate such processes.

**Figure 2:**
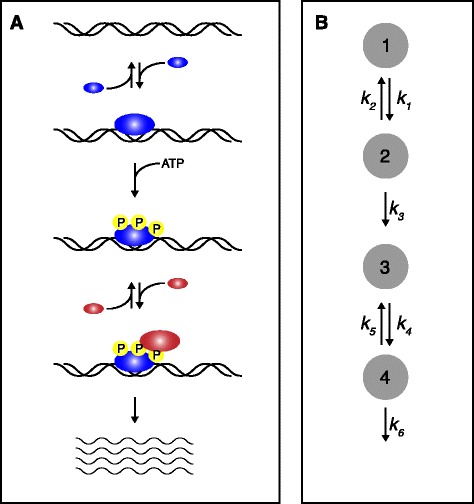
**Representing arbitrarily complex gene regulatory mechanisms with a graph-based framework. (A)** Cartoon schematic for a simple transcriptional regulatory mechanism involving equilibrium (binding and dissociation) and non-equilibrium energy consuming (phosphorylation) steps. Each step is a particular molecular arrangement that can be thought of as a ‘microstate’ which has a certain probability of being occupied based on the interactions, rates and concentrations of the molecules. **(B)** Graph depiction of the cartoon gene regulatory mechanism. Each node represents a microstate, the edges the interactions/reactions, and the edge labels are the rates.

In this issue of *BMC Biology*, Ahsendorf *et al.* [1] describe a general framework to incorporate non-equilibrium events into models of gene regulation. As suggested by the word framework, the approach outlined in the paper is not a computational model that comes as a package of MatLab or Python code. Rather, it is a way of formally abstracting gene regulation as a graph. A graph consists of nodes and edges: the nodes represent ‘microstates’ and the edges represent transitions between microstates. Microstates, in the case of gene regulation, are particular molecular arrangements that can be thought of as discrete steps in the process. For example, imagine the case where there is a transcription factor (TF) that binds to a promoter and must be phosphorylated to be able to recruit RNA polymerase to initiate transcription (Figure 2). In this example, there are four microstates related to the promoter: 1) naked promoter, 2) TF bound to the promoter, 3) phosphorylated TF bound to the promoter and 4) phosphorylated TF bound to the promoter with RNA polymerase. Each of these microstates is a node in the graph; the edges that connect them are the reversible interactions (binding and dissociation) and irreversible reactions (phosphorylation and transcriptional activation) that drive the transcriptional process; and the labels of the edges are the on, off and reaction rates. As the authors show, the probability that each microstate is occupied can then be calculated over time using standard differential equations and linear algebra without any requirement for thermodynamic equilibrium.

To demonstrate the utility of this framework, the authors apply their approach to three case studies of gene regulatory models in published literature to gain insight into the effects of non-equilibrium processes. In the first application, to transcription driven by nuclear hormone receptors, the authors show that, despite the known role of non-equilibrium ubiquitination in hormone receptor regulation, the non-equilibrium effects remain hidden and first order equilibrium models suffice. Next, the authors use their framework to cast doubt on the stability of inherently bounded chromatin domains that was suggested by numerical simulation - any stable chromatin mark that can be propagated will eventually lead to unbounded spreading unless non-equilibrium dissipative mechanisms are involved. In this case, the framework allowed first principle-based conclusions for the effects of non-equilibrium processes to be drawn without numerical simulation. Finally, by applying their framework to the regulation of the yeast *PHO5* promoter, the authors show clear effects of non-equilibrium processes in the complexities of the gene regulation function that have yet to be understood molecularly or revealed experimentally.

As increasingly sophisticated experimental techniques continue to reveal novel complexities involved in eukaryotic gene regulation, theoretical formalisms that allow us to model gene expression need to keep pace. Techniques such as ChIP-seq have revealed non-random localization of histone modifications, massive complexes of transcription factors termed ‘super-enhancers’ and poised/bivalent developmentally regulated genes [5,6]. More recently, experimental techniques enabled by high-throughput sequencing technology that map the three-dimensional architecture of the genome, such as Hi-C and ChIA-PET, have revealed long-range interactions and cohesin-bounded megabase-scale chromatin domains [7]. Needless to say, these structures and organized domains are established and maintained through non-equilibrium processes. The graph-based framework and operations described by Ahsendorf *et al.* provide a theoretical toolkit to formally accommodate such processes, and will hopefully enable us not only to more realistically model gene regulation, but also to gain insight into the currently opaque functions of non-equilibrium processes involved in chromatin organization and regulation.
